# Osteoarthritis: Pathogenesis, Animal Models, and New Regenerative Therapies

**DOI:** 10.3390/jcm12010005

**Published:** 2022-12-20

**Authors:** Tomasz Szponder, Michał Latalski, Anna Danielewicz, Katarzyna Krać, Aleksandra Kozera, Beata Drzewiecka, Dominika Nguyen Ngoc, Dominika Dobko, Joanna Wessely-Szponder

**Affiliations:** 1 Department and Clinic of Animal Surgery, Faculty of Veterinary Medicine, University of Life Sciences, 20-612 Lublin, Poland; 2 Children’s Orthopaedic Department, Medical University of Lublin, 20-093 Lublin, Poland; 3 Students Research Group of Veterinary Analysts, Sub-Department of Pathophysiology, Department of Preclinical Veterinary Sciences, University of Life Sciences, 20-033 Lublin, Poland; 4 Sub-Department of Pathophysiology, Department of Preclinical Veterinary Sciences, Faculty of Veterinary Medicine, University of Life Sciences, 20-033 Lublin, Poland

**Keywords:** osteoarthritis, articular cartilage, chondrocyte, animal models, regenerative medicine

## Abstract

Osteoarthritis (OA) is a chronic, progressive, multifactorial disease resulting in a progressive loss of articular cartilage structure and function that is most common in middle-aged and older patients. OA is involved in the loss of extracellular matrix and cartilage as well as cell number decreases within the matrix, especially in the further stages of the disease. The immune system plays a pivotal role in the pathomechanism of this condition. Both humoral and cellular mediators contribute to cartilage destruction, abnormal bone remodeling, synovitis, and joint effusion. The increasing prevalence of this disease has led to a growing interest in using animal models as the primary way to broaden the knowledge of the pathogenesis of OA and possible therapies at each stage of disease development. This review aims to describe the signs, pathogenesis, and classification of OA as well as discuss the advantages and disadvantages of some animal models. The currently used treatment methods include mesenchymal stem cells, exosomes, gene therapies, and blood-derived products. In addition, exogenous growth factors, platelet-rich plasma (PRP), platelet lysate, and autologous conditioned serum (ACS) are discussed with the application of tissue engineering techniques and biomaterials.

## 1. Introduction

Osteoarthritis (OA) is a chronic progressive disease with a complex multifactorial etiology. The disease results in a progressive loss of articular cartilage structure and function, especially in middle-aged and older patients. OA is considered one of the most common musculoskeletal diseases affecting the joints of the knee, hips, or hands and one of the most frequent causes of the disability. This joint disorder affects millions of people worldwide [1,2]. Currently, 25% of people over the age of 21, or more than 50 million people in the United States, are affected by OA, whereas in Europe it is about 100 million people. OA occurs due to the progressive and continuous destruction of articular cartilage due to different primary and secondary causes. The main clinical signs include chronic pain, joint instability, stiffness, and joint space narrowing, which is confirmed by radiography. Although OA mainly affects the elderly, sports-related injuries can lead to post-traumatic OA (PTOA), regardless of age. It has been shown that, despite careful postoperative care, PTOA occurs in 20% to 50% of patients [3,4].

OA is also a severe problem in veterinary medicine, especially in horses [5,6,7,8] and dogs in the form of hip OA [9]. Data show that as many as 20% of dogs over one year of age show some degree of degenerative changes in the joints; in cats over 12 years of age, this value is 90% [10].

The pathological changes in the surrounding subchondral bone and synovium are involved in OA. Osteoarthritic cartilage is confirmed by changes in biochemical indicators and the loss of the extracellular matrix, the loss of cartilage, and a decrease in the cell number within the matrix, especially in the further stages of the disease [11,12,13]. Several risk factors associated with OA have been described, including genetic predisposition, aging, and obesity, but the pathogenesis of OA remains unclear. In OA, articular cartilage and subchondral bone destruction lead to progressive locomotor disability and pain [14]. The treatment of OA mainly includes alleviating pain, reducing stiffness, maintaining functional capacities, and improving quality of life [15]. Despite of the high socioeconomic impact of OA, the available therapeutic options are minimal. Currently, there is no effective treatment to regenerate joint tissues, and OA therapy is restricted to alleviating symptoms until the joint is surgically replaced [14]. Therefore, tremendous efforts have been put into introducing more effective strategies [16].

The prevalence of this disease has led to increased interest in using animal models as the primary way to learn about the pathogenesis of OA and possible therapies at each stage of disease development. This review aims to describe the signs and pathogenesis of OA and discuss some animal models’ advantages and disadvantages. In addition, this review discusses the currently used treatment options, including, among others, mesenchymal stem cells, exosomes, gene therapies, some blood-derived products such as exogenous growth factors, platelet-rich plasma (PRP), platelet lysate, and autologous conditioned serum (ACS) as well as the application of tissue engineering techniques and biomaterials.

This review is an approach to facilitate the recognition and understanding of pathomechanisms, the selection of the best experimental model for translational medicine, and the planning of effective causal treatments, including the recent achievements in regenerative medicine.

## 2. Pathogenesis of OA

Some components, such as the meniscus, articular cartilage, subchondral bone, and synovial membrane, provide sufficient support in the healthy joint. The meniscus provides several functions, including load bearing and shock absorption. This structure consists mainly of water and components of the extracellular matrix (ECM) such as collagen and proteoglycans. The articular cartilage provides a surface for the movement of the synovial joint and consists mainly of proteoglycans and type II collagen. In contrast, the main component of the subchondral bone is a mineralized type I collagen. The synovial membrane (synovium) produces the synovial fluid, composed of lubricin and hyaluronic acid (HA), which lubricates the joint and nourishes the articular cartilage. The synovium consists of two types of synoviocytes: activated macrophages and fibroblasts, which produce the synovial fluid components [17].

Different abnormalities in the function of these structures have been found to promote OA [17]. This condition is characterized by alterations in the cartilage, bone, synovium, synovial fluid, ligaments, tendons, and joint capsule [18,19]. During the early stages of OA, the cartilage surface remains intact because of increased compensatory mechanisms [4]. However, during the progression of OA the molecular composition and organization of the ECM are changed first [20]. In addition, repetitive mechanical abrasions lead to degenerative alterations in the meniscus, with a loss of type I and type II collagen, whereas proinflammatory cytokines disrupt cartilage matrix homeostasis. Thus, the importance of the inflammatory mechanism in the initial stages of the disease has been confirmed [17,21,22].

The articular chondrocytes have little regenerative capacity and low metabolic activity in normal joints. They present a transient proliferative response and hypertrophic differentiation in increased matrix synthesis, which is attempted to initiate repairs in response to pathological processes. The altered composition and structure of the cartilage cause the stimulation of chondrocytes to produce larger amounts of mediators involved in cartilage degradation. Then, articular chondrocytes undergo apoptosis, and the articular cartilage is finally wholly destructed [15].

As a result of the increased protein catabolism, there is an imbalance in the synthesis of collagen and proteoglycans, and collagen fibers stop associating with proteoglycans. This causes the loss of the net woven fibers in the cartilage, weakening its strength. As a result, gaps are formed on its surface. At the same time, inflammatory mediators affect the surrounding tissues, leading to changes in the subchondral bone and the synovium of the joints. Cartilage undergoes fibrosis in regions affected by mechanical stress, with bone sclerosis and a thickening of the synovium and joint capsule. Cartilage fragments from degenerated articular surfaces induce the inflammatory process of the synovium, disrupting the synthesis of synovial components, such as HA, making the synovial fluid less viscous and elastic and eliminating its ability to moisturize cartilage. In response to tissue damage, proinflammatory mediators such as interleukin-1 (IL-1) and tumor necrosis factor (TNF-α) are released, stimulating the production of joint proteases. The chronic inflammatory process sensitizes the receptors exposed due to the disease, and their constant stimulation leads to further sensitization.

Consequently, even a standard stimulus can provoke a sensation of pain. In addition, the repair capacity of cartilage is limited due to its low mitotic activity, lack of vascular and nervous supply, and immobility [18,19].

### 2.1. The Origin of Primary Mediators of the Inflammatory Response in OA

**Figure 1:**
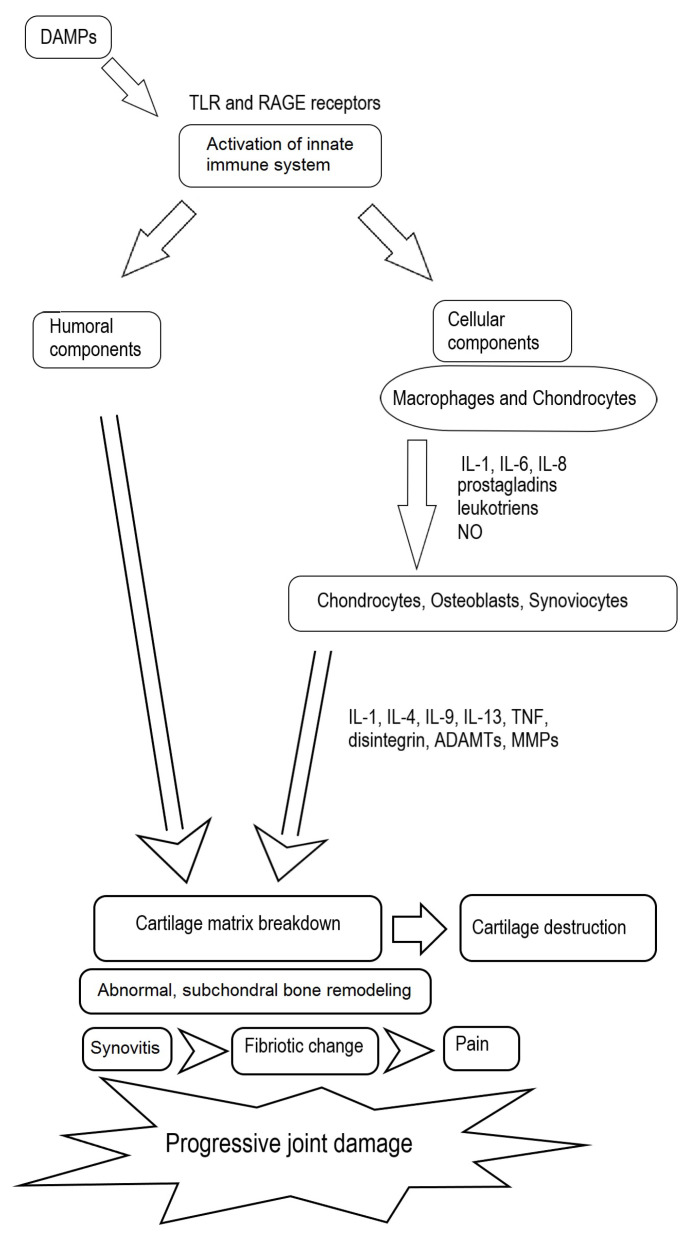
Joint damage mechanisms.

The immune system plays a crucial role in the pathomechanisms of OA. Both humoral and cellular mediators contribute to cartilage destruction, abnormal bone remodeling, synovitis, and joint effusion [23] (Figure 1). The innate immune system has a significant role in this process. Damage-associated molecular patterns (DAMPs) are endogenous molecules that include products of ECM damage, alarmins, free fatty acids, and debris from dead cells. DAMPs activate the innate immune system by interacting with pattern-recognition receptors (PRRs) such as Toll-like receptors (TLR), especially TLR2 and TLR4, mediating catabolic responses, and the receptor for advanced glycation end products (RAGE) present on the surfaces of immune cells. Moreover, complement system activation is implicated in the early stages of OA. The released products induce chondrocyte apoptosis or cause them to produce matrix-degrading enzymes, proinflammatory mediators, and additional complement effectors that promote joint damage [24].

An evaluation of the cytokines and chemokines involved in OA progression revealed the upregulation of IL-1, IL-6, and IL-8. These mediators act in both autocrine and paracrine manners. They stimulate macrophages and chondrocytes to release proteases and eicosanoids, such as prostaglandins and leukotrienes, as well as nitric oxide (NO). Moreover, this activity in the cartilage results in the induction of the catabolic processes, the inhibition of matrix synthesis, and the promotion of apoptosis [25].

In normal adult cartilage, chondrocytes synthesize matrix components very slowly [26]. During OA, the hypertrophic chondrocytes lose the ability to form new cartilage matrix. As a result, the subchondral bone undergoes abnormal remodeling in the interface between the bone and calcified cartilage. The released metalloproteinases (MMPs) degrade the matrix and, thus, articular cartilage. This leads to the formation of subchondral cysts and osteophytes to correct the joint instability. Abnormal bone remodeling also results in subchondral sclerosis, which may either occur late in the disease process or cause OA [17].

The release of some products, including other cytokines, such as IL-1, IL-4, IL-9, IL-13, and TNF-α, and degradative enzymes, such as a disintegrin, thrombospondin-like motifs (ADAMTs), and MMPs, by chondrocytes, osteoblasts, and synoviocytes triggers these processes [17]. The production of IL-1 by the activated chondrocytes induces the synthesis of MMPs, namely MMP-1, MMP-3, and MMP-13, and the simultaneous inhibition of the synthesis of key components of the ECM, such as proteoglycans, aggrecan, and type II collagen. This is accompanied by the amplification of proinflammatory cytokines such as TNF-α, IL-6, and IL-8, which enhances the cartilage matrix degradation in the catabolic cascade, further stimulating articular chondrocyte destruction [26].

The source of pain in the course of OA is involved in the inflammation of the synovial membrane, called synovitis, which undergoes progressive fibrotic changes. Pain may also be caused by the remodeling of the subchondral bone with rich innervations and peripheral neuronal sensitization. In older individuals, some other factors should be taken into account, namely the increased production of proinflammatory cytokines by chondrocytes and the role of the accumulation of advanced glycation end products (AGE). These products bind to receptors on chondrocytes, leading to the release of many proinflammatory mediators. Another factor involved in OA progression appears to be obesity. Some cytokines released from adipose tissue and the infrapatellar fat pad in the knee (adipokines) are involved in the degradation of articular cartilage [17,27].

Previous studies described OA as a process involved in local inflammation. However, some recent research indicates that inflammatory events in joints could be reflected in the plasma and circulating neutrophils, confirming systemic inflammation in OA patients and in the rabbit OA model. Therefore, OA should be considered a systemic musculoskeletal disease [27,28,29,30].

### 2.2. The Components of the Cellular Response and Cells Involved in OA

Monocytes appear to be involved in the subchondral bone and synovium activation in the pathogenesis of OA. Cytokines and chemokines released by monocytes are found at increased concentrations in the synovial fluid of osteoarthritic joints. In animal models, the depletion of synovial macrophages derived from monocytes decreases osteophyte formation and cartilage destruction. These data suggest that monocytes, monocyte-derived osteoclasts, and synovial macrophages participate in the pathogenesis of OA. Therefore, reductions in monocyte activation and joint recruitment may be beneficial in OA [23].

Osteoclasts derived from monocytes in the appropriate microenvironment can contribute to cartilage degradation. These cells release vascular endothelial growth factor (VEGF), TNF-α, IL-1b, IL-6, and chemokines, stimulating the vascularization of the synovium and the recruitment of circulating white blood cells. The thickening of the synovial membrane is involved in migrated monocytes, which differentiate into synovial macrophages. The components of the synovial fluid may additionally activate the subchondral bone through osteochondral lesions [23].

Chondrocyte hypertrophy has been described in both human and animal OA. Hypertrophic changes trigger chondrocyte activity and play a key role in the development of OA. The destruction of cartilage occurs through many mechanisms, especially the increased production of proteases such as MMP13. These alterations drive the disease-strengthening loop and provoke tissue degradation, remodeling, and finally calcification [31].

Neutrophils, present in the synovial fluids of patients suffering from joint disorders, have the highest impact on cartilage degradation. In joint inflammation, neutrophils invade the joint space and release different cartilage-damaging products, such as reactive oxygen species (ROS) and released proteolytic enzymes [32]. In addition, some neutrophil products contribute significantly to developing joint inflammation, pain, and peripheral neuropathy, suggesting their potential as therapeutic targets [30,33].

Macrophages are recognized as key factors in inflammatory joint disorders. Depending on their phenotypes, M1 or M2 macrophages can act as either pro- or anti-inflammatory cells. In the presence of IFN, macrophages are polarized toward an M1 proinflammatory phenotype with the release of TNF-α and IL-1b. This activity leads to the exacerbation of the inflammatory process and cartilage destruction. During the early inflammatory processes, the progression of OA has been noted and has led to new therapeutic strategies based on the stimulation of a proper anti-inflammatory cellular response to prevent cartilage destruction [24]. Recently, some research was conducted on the possible modification of macrophages in vitro after stimulation with blood-derived products, such as antimicrobial peptides (AMP), PRP, and microvesicles. The obtained results are promising and possible to extrapolate to a clinical application after clinical trials [34,35].

## 3. Classification of Osteoarthritis

The heterogeneous etiology of OA poses challenges for its classification and the introduction of efficient treatments. OA is typically classified into primary (idiopathic) and secondary OA based on the etiology. Primary OA is a naturally occurring condition due to degenerative changes in the joint. It is classified into localized OA, which affects one joint, and generalized OA, which affects more joints [17]. Idiopathic OA might occur naturally, such as in genetically modified mice [19]. Secondary OA, in turn, is involved in different causes and risk factors, classified as systemic risk factors (gender, age, diet, congenital diseases, and metabolic disorders or bone diseases) and local risk factors (injury and physical activity/sport). For these reasons, one universal treatment cannot be applied to all patients suffering from OA [2,17].

Five phenotypes of OA have been proposed based on the causes, symptoms, and possible treatment options:

1. Post-traumatic OA (PTOA) is caused by acute or repetitive joint injury. The prevention of injuries caused by falling in older adults; the usage of preventative measures, such as braces for athletes; and the prevention of surgical interventions such as meniscectomies should be introduced [17].
2. The metabolic phenotype involved in obesity is the effect of increased loading on weight-bearing joints and some individual metabolic factors, such as type 2 diabetes mellitus, dyslipidemia, and hypertension, and may increase the risk of obesity-induced OA. The mechanical overload and the systemic inflammation caused by a release of mediators from components of adipose tissue (e.g., adipokines, free fatty acids, and ROS) are the leading causes of the increased incidence and prevalence of OA in obesity. Estrogen-deficiency-related OA should also be taken into account. In this type of OA, a weight loss exercise program and a hormone therapy for menopause-related OA should be considered [17,36].
3. The aging phenotype is similar to PTOA. It is a naturally occurring phenotype that is strongly involved in advanced aging. The treatment for this phenotype could be targeted to inhibit AGEs and the proinflammatory cytokines released from senescent chondrocytes [17].
4. The genetic-based phenotype involves hereditary factors that affect the course of OA through different mechanisms. Therefore, the treatment strategy could be based on specific targets for gene or drug therapy [17].
5. The pain phenotype is involved in the pain in OA caused by inflammation and abnormal bone remodeling in the joint, and it needs the development of anti-inflammatory and pain medications [17]. Animal models are a valuable approach to studying the complexity of OA pain. However, one of the major problems is the translatability of animal models to humans. No model fully recapitulates human OA, and no pain test used in animals can fully reflect the human disorder, which complicates preclinical research [37].

Additionally, it should be mentioned that, apart from this classification, there are some novel methods and approaches for phenotype definition, especially those based on magnetic resonance imaging (MRI) of bone marrow lesions (BMLs) [16].

## 4. Classification and Description of Animal Models

### 4.1. Features of an Ideal Animal Model and Common Animal Models Used for OA

The main goal of animal models is to reproduce human disorders, and due to the heterogenicity of human OA, different models should be used [19]. In order to choose the best animal model for the study of OA, the course of the disease should be consistent with that in humans, reproducible, and included in specific time frames. The induced disease should be progressive so that it is possible to examine its early, middle, and late stages and to observe the effects of treatment. In addition, the model should be mammalian, relatively cheap (for economic reasons), easy to maintain, and large enough to perform all necessary assays, such as X-ray, MRI, histopathology, or synovial fluid analysis. Pain testing is also essential, and the pain should be responsive to NSAID and opioid therapies. Finally, the treatment must be similar in human and animal models [18].

**Figure 2:**
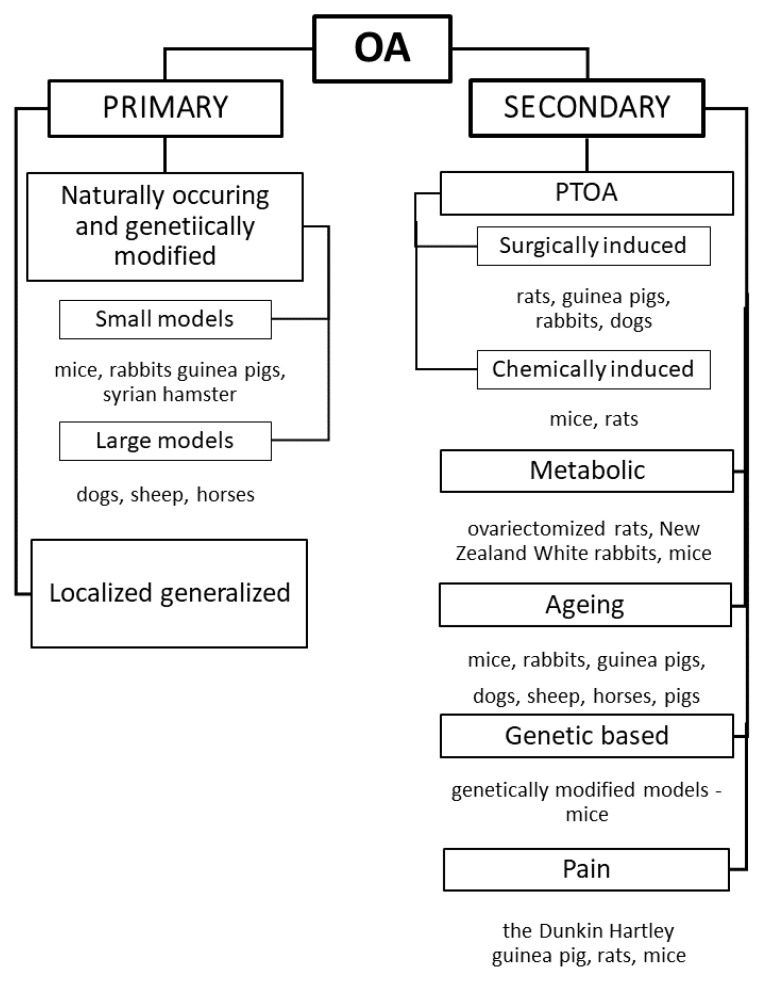
Osteoarthritis classification and models.

Different species of animals, such as mice, rats, rabbits, guinea pigs, dogs, sheep, pigs, and horses, have been used as OA models. The choice of each animal depends on several factors, including the design and time course of the experiment, husbandry costs, ease of handling, and outcome measurements [17,19]. The most widely used models are small animals because of the easy access to animal facilities. Large animals are less commonly used because of the costs [19] (Figure 2).

### 4.2. Animal Models with Primary OA (POA)

#### 4.2.1. Naturally Occurring Primary OA

Naturally occurring OA has been confirmed in aged mice, rabbits, guinea pigs, dogs, sheep, and horses [37]. The typical animal model to study naturally occurring OA is the Dunkin Hartley guinea pig, which reflects POA in humans. This model has some advantages; the rapid growth to maturity and the development of lesions similar to humans enables its possible use in evaluating pathomechanisms and therapeutic applications. Moreover, the guinea pig is an excellent naturally occurring model to study the inflammatory process in the joint. Spontaneous OA changes are shown in about 50% of 3-month-old guinea pigs, weighing about 700 g, as the symmetrical defects on the medial tibial plateau in places not protected by the meniscus lead to focal chondrocyte death and the loss of proteoglycan. At the age of 6 months and weighing 900 g, 90–100% of the animals show lesions. In addition, 9-month-old animals have mild to moderate defects in the middle tibial cartilage, mild degeneration of the condyle, and tibial osteophytes [17].

Syrian hamsters are other commonly used experimental animals that develop spontaneous OA caused by various factors, such as the dislocation of the patella, valgus and varus abnormalities, and inherited genetic mutations, including a type II collagen gene mutation. The first signs of cartilage degeneration were shown in 6-month-old animals [38]. The degenerative changes may be severe, with full-thickness cartilage loss and the presence of large osteophytes before the animals are 15 months old. In hamsters, the cartilage has relatively few cell layers compared to the larger species. Therefore, the scoring of cartilage lesions can be challenging. However, if relatively simple scoring systems are used, reproducible data can be obtained [38].

In some strains of mice, such as STR/ort and C57BL/6, the genetic predisposition to developing spontaneous OA, even in very young animals (18 weeks of age), was confirmed. Other mouse strains, such as CBA, have been described as resistant to the development of OA (as a negative animal model) [39].

Dogs are known as natural animal models in preclinical trials of therapeutic interventions [10,17].

Among the models used to study the pathogenesis of OA, horses are distinguished by their large size, which makes it easier to observe damage in the joints and the thickness of cartilage. It is similar to that of humans, which also contributed to the increased interest in horses as a research model for the study of articular cartilage repair, osteochondral defects, and bone remodeling. The obtained results could serve to develop and treat these changes in humans, especially in PTOA. Due to their use in sports, they are more prone to injuries resulting from the application of high mechanical force affecting the articular surfaces, so it is possible to observe the development of post-traumatic OA [17]. Severe decreases in joint function, pain, joint enlargement, and deformity are observed in horses with OA. It refers to one joint (monoarticular) or multiple joints (polyarticular). The etiopathogenesis of OA is incompletely understood. However, initial joint injury is a well-documented risk factor for the development of this disorder. Moreover about 12% of all OA cases may be involved in previous joint trauma due to altered biomechanics that resulted from a higher risk for progressive joint degeneration [5,6,40,41].

Sheep are the model for the evaluation of early cartilage changes in the course of OA. This animal model can be used to study meniscus changes and related surgical techniques due to its anatomical similarity to humans [17].

In the end, it should be mentioned that spontaneous OA has been reported in the knee joints of non-human primates. However, the varying severities of lesions and the difficulty in obtaining sufficient numbers of primates for reliable studies probably preclude the general use of this model [38].

#### 4.2.2. Genetically Modified Animal Models

Genetic engineering explores gene knockouts to determine the genetic factors involved in the predisposition to OA [39] Among the major advantages of the mouse as an animal model in OA studies is the ability for the genetic modification or preparation of specific strains, particularly those susceptible or resistant to OA. In the case of knockout mice, lacking some proteases or collagen type IX alpha one gene inactivation could make them resistant to developing OA [17] STR/ort mice can be used to show a correlation between OA and chondrocyte metabolism, and Col2a1 knockout mice have a higher incidence (60–90%) of natural OA than wild-type mice [17,39].

Although genetic engineering plays a crucial role in understanding the mechanisms of OA and the impact of genetics on the development of OA, the use of these models in the development of appropriate treatment options is limited [17,39].

### 4.3. Secondary OA Animal Models

#### 4.3.1. Post-Traumatic OA: Non-Invasive Animal Models

In experiments based on animal models, the procedure’s success also depends on the surgical procedure’s reproducibility on all animal subjects. Some of these problems can be resolved using non-invasive models. In the case of these models, mechanical injury is performed through physical impact without damage to the skin. This injury causes changes similar to those in surgically induced animal models and can be created with higher precision than in the more invasive models [17].

#### 4.3.2. Post-Traumatic Osteoarthritis: Induced/Invasive Models

Invasive models have been used to study the effects of different treatments of OA. They are classified into surgically induced and chemically induced models. The rapid induction of OA in these models ensures a short time frame for the experiment. However, the induced models do not reflect humans’ natural degenerative changes. Despite these limitations, surgically induced models have been used to study the pathogenesis of PTOA, mainly involving subchondral bone changes [17].

#### 4.3.3. Animal Models for Surgically Induced OA

Rats

The progressive degenerative cartilage changes characterized by chondrocyte and proteoglycan loss, fibrillation, osteophyte formation, and chondrocyte proliferation are evoked rapidly by unilateral medial meniscal tears. The procedure is based on the surgical transection of the medial collateral ligament, and the meniscus is cut at its narrowest point without damaging the tibial surface [38].

Guinea Pigs

In guinea pigs, the medial aspect of the knee joint is preferentially loaded. Therefore, the surgical procedure should be performed on the medial side to induce the desired pathological changes [38].

Rabbits

**Figure 3:**
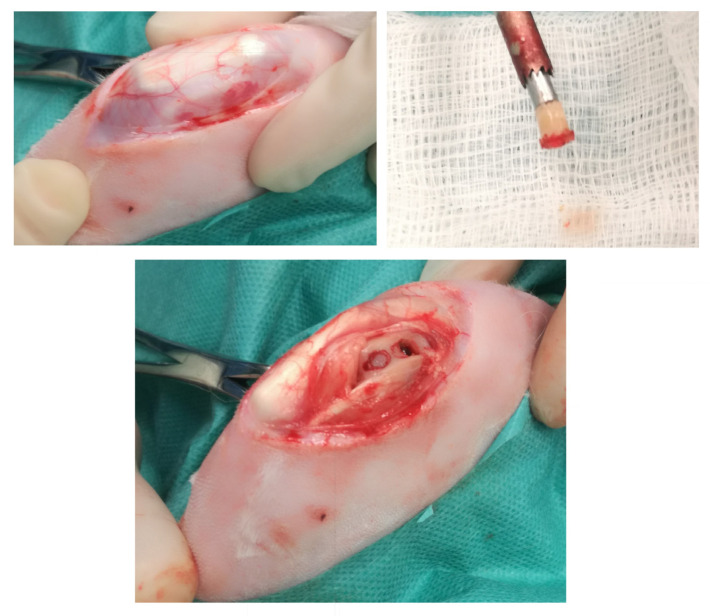
Autologous osteochondral grafting in a rabbit model.

The rabbit is a standard model for the evaluation of human OA and has been used for assessing cartilage repair and the treatment of osteochondral defects [19,30,38,42,43,44]. A meniscectomy, or the partial removal of the meniscus, in New Zealand White rabbits causes damage similar to that found in humans with OA. These lesions could be evoked by partial meniscectomy. Performing this procedure on the medial aspect of the joint generally causes relatively mild to moderate degenerative changes. This model has been used extensively to test potential chondroprotective treatments. A partial lateral meniscectomy evokes a consistent degenerative focus involving approximately half of the lateral tibial plateau and femoral condyle. The termination of the studies 6 weeks postsurgery is adequate for evaluating the effects of compounds on marked to severe focal chondrocyte loss, proteoglycan loss, and fibrillation [38]. Research on the rabbit model proved helpful in expanding the knowledge of OA autografting and allografting as an increasingly important treatment for human articular cartilage injury (Figure 3). In addition, the rabbit model of OA has allowed the study of the immune response involved in the osteochondral tissue, which is essential to repair efficiency, and the potential mechanisms of graft success or failure [30].

Dogs

In dogs, the removal of the anterior part of the meniscus develops moderate changes in femoral cartilage. One of the main advantages of using dogs as models is a much slower formation of cavities in cartilage than in rats or rabbits subjected to removing the side of the meniscus. Surgery to cut a dog’s anterior cruciate ligament causes a destabilization that causes damage similar to that caused by OA and over time causes the appearance of OA itself. This procedure provides an opportunity to observe the slow development of OA. The use of beagles for OA models offers the opportunity to obtain data from research on a species commonly used for toxicology tests. It is essential to house the dogs in large runs that allow the opportunity for movement. The observed alterations are much milder and highly variable when housing experimental dogs in stainless-steel cages with intermittent exercise. Moreover, the induction of minimal trauma is essential for better and faster recovery. In dogs, in contrast to rodents, the gait/load-bearing patterns are altered after surgical procedures, and the duration of the disturbance depends on the extent of the instability [38] (Table 1).

#### 4.3.4. The Role of Animal Models in Regenerative Therapies

Preclinical studies of OA regenerative therapies require the use of animal models to investigate their effectiveness. The use of such animal models of the disease allows research to be conducted in a way that would be impossible in humans, e.g., by performing invasive procedures, providing more accurate knowledge about the disease being studied. However, the complexity of the disease process arising in these organisms may make it difficult to understand its course. For this reason, it is sometimes easier to analyze diseases caused in a simplified system in which the individual parts of the pathological process are isolated. In vitro studies, on the other hand, do not reflect the characteristics of the actual articular space, biomechanics, or the influences of surrounding tissues. The most commonly used animal models are mice and rats due to their low maintenance costs and relative ease of handling and genetic manipulation, making them more suitable for synthetic and genetically related primary OA models. However, due to their small sizes, it is difficult to compare them with human biomechanics. Therefore, larger animal models (e.g., goats, sheep, and horses) with much larger knee joints, comparable in size to humans, can be assessed using arthroscopy and MRI. However, unlike the previously mentioned mice and rats, these larger animals are not as prone to developing spontaneous OA as quickly and usually require surgical or dynamic induction to produce degenerative joint changes. Larger animal models are also more heterogeneity, as in humans, and have complex genetic and physiological interactions with the environment, making them ideal for assessing the safety and efficacy of new therapies. Choosing the most suitable animal model for testing is crucial for ensuring a successful experimental outcome. Recent developments in biomedical sciences have provided scientists with alternative approaches to tissue repair. Nevertheless, this branch of science requires further progress in order to achieve the most favorable strategies and solutions [39].

#### 4.3.5. Chemically Induced Models

Chemically induced models can be used to evaluate the effects of drugs on inflammation and/or pain and are mostly obtained by injecting a toxic or inflammatory compound into the joint. Some substances, such as papain, sodium monoiodoacetate (MIA), quinolone, and collagenase, are applied to induce OA in animals. These procedures eliminate the need for surgery and avoid possible infection. Although chemically induced models are less invasive, in chemical models the pathomechanisms of development are different and do not correlate with PTOA. Therefore, their usefulness is mainly restricted to evaluating the mechanism of pain and investigating drug therapy. The most commonly used compound is MIA acting as inhibitor of glyceraldehyde-3-phosphate dehydrogenase in the Krebs cycle, leading to such alterations as the death of chondrocytes, osteophyte formation, and articular cartilage degradation. As a result, acute inflammation and pain develop and last for seven days. Then, chronic changes develop. The MIA-induced model measures pain and drug efficiency to resolve the pain in animal models, mainly in mice and rats [17].

Another toxic compound, quinolone, is a potent broad-spectrum antibiotic that targets DMA gyrase (bacterial topoisomerase). It can simultaneously cause articular cartilage degeneration in growing animals connected with the loss of proteoglycans and chondrocytes and usually causes growth defects in children [17,38]. This occurs through its action on the epiphyseal growth plate; for this reason, its use is contraindicated in adolescents and pregnant women [17]. The administration of quinolones to guinea pigs induces a characteristic blister-like lesion in the midzone cartilage. Degenerative alterations develop over 24 h, including focal swelling, decreased toluidine blue staining, and chondrocyte death. In the end, the upper layers desquamate off, leaving an area of fibrillation. Cells and proteoglycans are lost, and these changes resemble the lesions seen in OA [38].

In the course of OA, the release of collagenase leads to the degradation of proteins in the articular cartilage. In this mechanism, in a chemically induced model, the intra-articular administration of collagenase caused type I collagen destruction, leading to the degradation of the collagen matrix in the tendons and ligaments and finally to joint instability [17].

## 5. Current Regenerative Therapies and Prospects

The traditional treatments for OA are limited to controlling the symptoms, but none of them can reverse the damage in the joint, and their use is associated with a high incidence of adverse effects. Evaluations of new OA treatments with higher effectiveness and fewer side effects are underway. However, the treatment of damaged articular cartilage remains one of the major challenges in regenerative medicine. It should be underlined that regenerative therapy maintains the potential for repairing destroyed tissues to restore their original structure and function [2,45].

### 5.1. Examples of Pharmacologic Strategy

Using antirheumatic and other anti-inflammatory strategies appeared to be a promising option for the treatment of OA, after success in preclinical trials. However, they have not been fully tested in clinical studies [17]. Since the cytokines IL-1b and TNF-α appeared to be the most important in cartilage destruction, specific inhibitors of their activity, such as a recombinant human IL-1 receptor antagonist (IL-1ra) have been introduced. Another approach uses monoclonal antibodies against IL-1 or the type I IL-1 receptor (IL-1RI). This is a safe strategy in the early phase of OA to inhibit inflammation and promote cartilage repair. MMP13 and Adamts-5 are the main matrix-degrading enzymes that play a key role in the development of OA. Therefore, the inhibition of matrix degradation also appeared to be effective in the treatment of OA in animal models [45].

In articular cartilage, many growth factors are responsible for the development and maintenance of the homeostasis of articular cartilage. The imbalance may disrupt tissue repair, resulting in the decreased synthesis of ECM, tissue degeneration, and ultimately the erosion of the articular surface. Transforming growth factor beta (TGF-b)/bone morphogenetic protein, insulin-like growth factor-I (IGF-I), and fibroblast growth factors (FGFs) are considered crucial anabolic factors for cartilage repair. They stimulate chondrocytes for the synthesis of proteoglycans, aggrecan, and type II collagen, inducing cell proliferation, driving the chondrogenic differentiation of stem cells, and limiting the catabolic effects of proinflammatory cytokines. The overexpression of one of the growth factors, namely progranulin (PGRN), is involved in the stimulation of chondrocyte proliferation; PGRN also acts as a physiological antagonist of TNF-α signaling, potentially inhibiting cartilage degradation.

Growth factors such as platelet-derived growth factor (PDGF) and TGF-b as components of PRP, decreased IL-1b-induced NF-κB activation, a crucial pathway involved in the pathogenesis of OA. Moreover, TGF-b, delivered with calcium alginate to the sites of osteochondral defects in the rabbit knee enhanced the healing process. However, the local osteophyte formation has been noted in clinical trials. Therefore, caution is advised, as some growth factors promote stem cells dedifferentiation and the endochondral ossification process [46].

Some slow-acting chondroprotective compounds, such as glucosamine sulfate (GS), chondroitin sulfate (CS), hyaluronic acid (HA), and diacerein, are applied to enhance healing. Glucosamine decreased the activation of NF-κB in rat chondrocytes after treatment with IL-1b. In human chondrocytes, GS inhibited the NO generation induced by proinflammatory cytokines. In chondrocytes, CS diminished the increases in p38 MAPK and extracellular signal-regulated kinase 1/2 phosphorylation and decreased the NF-κB activation that inhibited the release of proinflammatory cytokines and enzymes such as phospholipase A2 (PLA2), COX-2, and inducible NOS (iNOS). HA is widely used and exerts significant chondroprotective effects, altering inflammatory mediators’ profiles and shifting the balance between matrix synthesis and degradation. CS, diacerein, GS, and HA caused pain reduction and the improvement of physical function with very low toxicity [45].

Metformin, the first-line drug for treating diabetes mellitus, appeared to be effective against OA. It diminished cartilage degradation by regulating the AMPK/mTOR signaling pathways, decreased the p16INK4a levels in OA chondrocytes, and enhanced the polarization of AMPK and the inhibition of mTORC1 in chondrocytes [13].

### 5.2. Blood-Derived Products

Different blood-derived products have become novel and attractive therapies because of the high concentrations of specific components that potentially affect tissue repair. These products are applied to OA joints. Platelet-rich plasma (PRP) is the product with the broadest application [14]. PRP is a product with significantly higher (generally 4–6 times) platelet and growth factor concentrations compared to the patient’s baseline concentration [47]. The adjunctive use of PRP produced a better effect in healing than some surgical procedures applied alone [43]. However, there is some controversy about its benefits, and it may not be efficient in stopping or reversing the degenerative process in the joints. The main disadvantage relates to the variability in its composition due to different production techniques and its relevant contents, especially of proinflammatory agents such as fibrin and leukocytes [14]. It should be mentioned that there are leukocyte-rich (L-PRP) and leukocyte-poor (PURE-PRP) variants of PRP with different contents [35]. The anti-inflammatory role of PRP was demonstrated in vivo in the porcine model of arthritis. After the intra-articular injection of PRP, the subsequent inflammatory response was attenuated. PRP may also improve the integration of an osteochondral graft at the cartilage interface and decrease degeneration in an in vivo rabbit model [46]. Conversely, in L-PRP the presence of leukocytes may trigger an inflammatory response [34,48].

Hyperacute serum (HAS), which maintains the properties of PRP, is a solution consisting of cells and fibrin-free serum, obtained after pressing out fibrin clots. In addition, HAS promotes cell viability and modulates inflammatory responses; therefore, it may be considered a possible therapeutic option for OA [14].

Platelet lysate (PL) is composed of many compounds, including growth factors such as PDGFs, VEGFs, TGF-β1, EGFs, and IGF-1, which interact with tyrosine kinase receptors, promote cell proliferation, and inhibit apoptosis. Other components are immunoglobulins, fibrinogen, and other coagulation factors. This product has many applications, including as a serum substitute and in improving wound healing and tissue repair [49].

Autologous conditioned serum (ACS) is a product for intra-articular application that was introduced to OA treatment in the mid-1990s due to its high concentrations of endogenous IL-1 receptor antagonist (IL-1Ra), cytokines, and growth factors, such as TGF-B, PDGF, IGF-1, IL-4, and IL-10 [47].

Another hemoderivative, namely an autologous protein solution, is a product with a high concentration of such anti-inflammatory compounds as IL-1 receptor antagonist, sIL-1RII, sTNF-RI, and sTNF-RII and is obtained from the serum of patients with confirmed OA [47].

Both products, autologous conditioned serum and autologous protein solution, are promising since they appear to be safe and utilize the patient’s serum, which is rich in cytokines and growth factors, so there is no immune response. They may be efficient in the treatment of knee OA [47].

### 5.3. Surgical Techniques

Some techniques to treat destroyed cartilage include arthroscopic lavage and debridement and bone marrow stimulation. Arthroscopic lavage removes loose cartilage, releasing inflammatory mediators and collagen debris that can cause synovitis and effusion. Chondroplasty, as the technique for cartilage debridement, is conducted to remove the free chondral flaps and fibrillated articular cartilage from the joint without damaging the intact tissue [2].

The technique of bone marrow stimulation relies on exposing the chondral defect to the bone marrow to create an environment that causes fibrocartilage healing. This technique is performed by microfracture and the subchondral drilling of cartilage with a high-speed drill through the trabecular bone. Blood perfusion results in the creation of a blood clot, which initiates the repair process with such constituents of cartilage as a mixture of hyaline and fibrocartilage. The microfracture technique allows the accurate debridement of damaged articular cartilage until the subchondral bone plate while maintaining a stable perpendicular cartilage edge. The defects are performed 3–4 mm apart with the use of an arthroscopic awl; then, the defect is filled with a fibrin clot, which creates the optimal environment for the pluripotential marrow cells to differentiate into a mature form. According to histological findings, after a microfracture procedure, a hyaline and fibrocartilage hybrid dominates the defect’s site [2].

Many surgical techniques have been developed to treat cartilage injuries, including autologous osteochondral transfer, autologous chondrocyte implantation, and osteochondral allograft transplantation [50].

#### 5.3.1. Autologous Osteochondral Transplantation

Autologous osteochondral grafting is a reliable method for treating injured articular cartilage. The main concerns of this technique are donor-site pain and morbidity in the injured joint. Another weakness is difficulty in restoring the shape of the femoral condyle [44]. Moreover, this intervention is for patients with an articular cartilage lesion less than 2–3 cm^2^ [51].

Autologous osteochondral transplantation is different from articular cartilage transplantation since it involves harvesting cartilage plugs from the margins of the knee joint and the intercondylar notch and transplanting them into the articular defect. This method is attractive because it is carried out as one procedure. However, it also has some weaknesses: the incomplete filling of the defect because of an insufficient amount of graft material and limitations due to the availability of grafts and the sizes and depths of defects (autologous osteochondral grafts) [30,52].

#### 5.3.2. Costal Cartilage Grafts

Mosaicplasty is a practical approach to resurfacing an osteochondral defect. However, limitations of this technique are a secondary injury at the donor site when autografts are acquired and the risk of disease transmission for allografts. Because costal cartilage is hyaline cartilage with active chondrocytes, autologous costal cartilage may be an option for articular cartilage in mosaicplasty. Costal cartilage is hyaline cartilage that presents phenotypic similarities to articular cartilage; therefore, it can be an autologous graft source in articular cartilage reconstruction. In the study of Du et al., the authors proved the feasibility of creating conditions suitable for newly forming osteochondral interfaces between costal grafts and host bones. The preliminary preclinical results were satisfactory. However, there are still some limitations. In the rabbit models, the high endogenous healing potential makes translation to clinical medicine difficult. Therefore, large animal studies using costal chondral grafts with clinically relevant sizes are necessary, as are more extended observations to investigate the changes in the costal cartilage post-transplantation [44].

#### 5.3.3. Osteochondral Allografts

The host’s immunologic response plays a crucial role in a successful osteochondral allograft implantation. Unlike other forms of allograft, fresh allogeneic osteochondral grafts do not match the human leukocyte antigen (HLA) or ABO blood group. Moreover, patients received no immunosuppressive treatment to prevent an immune response. However, the immunologic ramifications of this procedure remain an essential consideration and may be used to improve this treatment further to prevent graft rejection. It is well known that freezing or freeze-drying procedures reduce allograft immunogenicity; however, these methods of preserving allografts cause serious decreases in the viability of chondrocytes. As previously estimated, isolated chondrocytes and matrix components are immunogenic. However, the intact hyaline cartilage matrix is relatively immune-privileged. Findings show that the intact articular matrix protects the chondrocytes because of its structure, consequently making it difficult to be recognized by the immune system. Some studies demonstrated activating the recipient’s humoral immune system and underlined the potential relation between the host immune system and fresh osteochondral graft rejection. The antibody response to fresh, non-matched osteochondral allograft transplants in the knee appeared to be related to graft size. Graft success is multifactorial, and the impact of the immune response on clinical outcomes deserves further research [50].

Osteochondral allografting techniques can use fresh, cold-stored osteochondral allograft tissue, which is transplanted into the articular cartilage or osteoarticular defect. Fresh osteochondral tissue is used because it contains more significant numbers of viable chondrocytes. One can use this procedure to treat a wide spectrum of articular cartilage lesions, from focal chondral defects to established localized osteoarthrosis. Osteochondral allograft transplantation has proven success rates between 50% and 90% for treating focal chondral and osteochondral defects, osteochondritis dissecans, and post-traumatic, osteonecrotic, and bipolar lesions in the knee. Research has revealed several essential factors in graft survival, including cartilage cell viability after storage and adequate osseous support [50].

### 5.4. Cell-Based Therapy

Cell therapy for cartilage repair, introduced in the 1980s, has rapidly developed and currently offers a long-term solution to repair cartilage, alleviate symptoms, and delay OA progression. Cell therapy applies to both mature cells, such as chondrocytes, and stem cells [45].

#### 5.4.1. Chondrocytes

Autologous chondrocyte implantation/transplantation (ACI/ACT) is widely used in clinical practice. ACI collects a small portion of cartilage tissue from a healthy and less weight-bearing area during an arthroscopic procedure. The extracellular matrix is enzymatically removed, and chondrocytes are isolated and cultured in vitro. Then, the cultured chondrocytes are implanted into the damaged area of the cartilage.

During the most advanced procedure, called matrix-induced autologous chondrocyte implantation/transplantation (MACI/MACT), cultured chondrocytes are preseeded on a three-dimensional scaffold and trimmed to fit the defect size. Then, the obtained composite is implanted into the defect and fixed with fibrin glue. MACI shows evident advantages over classic ACI, as it reduces the surgical time, minimizes injury during fixation, and ensures long-term cell viability [45].

However, there are some limitations: a small number of available cells, multiple surgical procedures, in vitro chondrocyte dedifferentiation, and donor-site injury caused by the cartilage harvest. Therefore, mesenchymal stem cells (MSCs) are indicated as the potential cell source for this procedure. These cells can be easily obtained from tissues such as bone marrow, adipose tissue, synovial membranes, and others and have a high proliferation rate, chondro-differentiation capacity, and immunosuppressive activity [45].

#### 5.4.2. Mesenchymal Stem Cell (MSC) Therapy

MSCs are multipotent stem cells which possess the ability to migrate to different musculoskeletal tissues, especially to sites of injury, and undergo specific differentiations. Because of the chondrogenic differentiation potential, MSCs appeared to be promising candidate progenitor cell sources for cartilage tissue repair. MSCs are isolated from different tissues, such as adipose tissue, bone marrow, umbilical cord blood, placenta, synovium, periosteum, and muscle. MSCs can release a broad spectrum of soluble mediators with both immunoregulatory and regenerative properties [2]. MSCs also inhibit the destructive activity of MMPs mediated by TIMP. The joint repair function of MSCs was also confirmed using the caprine OA model. After an intra-articular injection of autologous MSCs, there was a significant regeneration of the medial meniscus, and implanted cells were observed in the newly formed tissue. In joints after treatment with MSCs, the degeneration of the articular cartilage, osteophytic remodeling, and subchondral sclerosis were reduced [45,53,54].

For all these reasons, MSCs appeared to be promising candidates for OA treatment. Currently, autologous MSCs are used predominantly because of the low risk of an immune response. However, since the capacities for the proliferation, differentiation, and survival of MSCs decrease with age, allogeneic MSCs from young donors are considered a better source for the treatment of OA [24]. Generally, MSCs implanted into the cartilage defect allow tissue repair and remodeling, especially with three-dimensional scaffolds and growth factors [2]. Moreover, allogeneic MSCs are less expensive to obtain and have a higher level of homogeneity [24].

#### 5.4.3. MSC-Derived Exosomes

Exosomes are a type of extracellular vesicle with a diameter of 30 to 140 nm that are secreted from many cell types, including lymphocytes, platelets, mast cells, dendritic cells, and tumor cells. Exosomes derived from MSCs have properties similar to those of their parental MSCs and can deliver more than 150 different miRNAs and more than 850 proteins, various DNAs, and lipids. In addition, they are able to distribute and gradually release anti-inflammatory factors for the treatment of OA and are involved in the activity of target cells through various pathways [24,55].

In light of experimental and clinical evidence, MSC-derived exosomes appeared to be a new and promising cell-free therapeutic strategy with advantages over MSCs, such as no risk of tumor genesis and low immunogenicity. They also play a key role in enhancing angiogenesis, which is crucial for tissue repair [55].

MSC-derived exosomes can be used as a natural drug carrier to increase the precision of administration and reduce the dose and possible side effects. However, the knowledge about treating OA with exosomes is still limited. Some weaknesses, namely an inefficient separation method, a lack of suitable visualization techniques, and the absence of specific biomarkers, need to be addressed [55,56].

#### 5.4.4. Pluripotent Stem Cells

Pluripotent stem cells have unlimited self-renewal and chondrogenic differentiation abilities. Therefore, they are considered a better source of cells for cartilage repair and OA treatment than chondrocytes or MSCs. Embryonic stem cells (ESCs) are derived from early mammalian embryos. Then, ESC chondrogenesis can be achieved in vitro after supplementation with growth factors. After the success of animal models in 2009, the US FDA approved a clinical trial with human ESCs. Another type of pluripotent stem cells that are induced to differentiate into various cell types, including chondrocytes, and are generated directly from adult cells are induced pluripotent stem cells (iPSCs). These cells are more applicable than ESCs, as they can be derived from more tissues, with a lower risk of immune rejection and less ethical controversy [45].

### 5.5. Tissue Engineering

Tissue engineering uses cells, scaffolds, and bioactive factors to enhance tissue mechanical properties and promote cell migration, attachment, proliferation, and differentiation. To date, tissue engineering has shown promising outcomes in treating cartilage defects, including OA [45].

Endogenous Cell Homing

Endogenous cell homing aims to change a suitable microenvironment to recruit and migrate the host cells from the circulation or tissues. This procedure is regarded as cost-effective and is technically less complicated than cell transplantation. Collagen type 1 scaffold containing stromal cell-derived factor-1 was previously used to create an in situ matrix environment. This microenvironment facilitated the migration and adhesion of endogenous MSCs, thereby promoting the self-repair of cartilage defects in a rabbit model. The plasmid-gene-activated osteochondral scaffold that could release TGF-β1 for the chondrogenic layer and BMP-2 for the osteogenic layer was also evaluated. Endogenous MSCs can be spatially controlled for simultaneous differentiation into chondro- and osteolineages within the scaffold. Therefore, as OA usually affects different joint tissues, this procedure may be applied in treating OA [45].

Cell-Based and cell-free scaffolds

Treatment with cell-based scaffolds involves tissue harvest procedures that are used in cell therapy. The cells are preseeded on the scaffold and implanted into the defect area with or without fixation. Many commercial products have been approved for scaffold-associated chondrocyte implantation, such as a bilayer collagen type 1/3 scaffold (Chondro-Gide, Geistlich Biomaterials, Wolhusen, Switzerland), a hyaluronan-based scaffold (Hyaff-11, Fida Advanced Biopolymers, Abano Terme, Italy), and a synthetic polymer scaffold composed of fibrin, polyglycolic/polylactic acid, and polydioxanone (BioSeed-C, BioTissue, Zürich, Switzerland). Cell-free scaffolds, in turn, were developed for a one-stage procedure. Scaffolds can be applied alone for the activation of endogenous cells or combined with bioactive products such as concentrated bone marrow or PRP [45].

### 5.6. Gene Therapy

Gene therapy aims to deliver nucleic acids to the target site using direct in vivo methods or ex vivo transducing by cells using some viral or non-viral vectors and enabling the spatiotemporal control and continuous synthesis of gene products [17]. Several preclinical studies have confirmed the method’s safety and efficacy and implicated its prospects. However, few clinical trials have been performed, and no gene products have been approved for OA treatment. Only TissueGene-C, based on TGF-β gene therapy, which uses retrovirally transduced allogeneic human chondrocytes overexpressing TGF-β1, has been clinically investigated in the United States and Korea [45].

### 5.7. Biomaterial Hydrogels for Cartilage Regeneration

There are some natural and synthetic biomaterials with the potential for developing hydrogels for cartilage regeneration. Polyethene glycol (PEG) is relatively inert and biocompatible. The incorporation of HA into PEG hydrogels improved the bioactivity of the PEG hydrogels [57]. Alginate hydrogels are used for cartilage regeneration, with properties for the promotion of cartilage ECM synthesis and chondrogenesis. The negative charges of the alginate structure provide the retention of newly generated aggrecan molecules. However, these materials have some limitations, including weak mechanical stability, slow degradation, and poor cell adhesion. In order to improve cell adhesion, the arginine–glycine–aspartic acid peptide sequence was immobilized in alginate scaffolds. Collagen hydrogels provide an advantage in cell aggregation and the initiation of a chondrogenic differentiation that reflects embryonic chondrogenesis [57].

The properties of HA were described in Section 5.1. HA is also used in hydrogels, especially in compositions with other components that modulate the chemical and mechanical properties of HA [57]. For example, Adamts-5 inhibitor and an HA hydrogel were combined to treat OA knee joints in a rat model and significantly decreased the progression of cartilage degeneration. In a mouse model, the application of a Syndecan-4-specific antibody prevented proteoglycan loss and cartilage destruction. Unfortunately, the clinical study with an MMP inhibitor (PG-116800) was terminated due to musculoskeletal toxicity without a clear benefit. Therefore, more preclinical studies of the safety and effectiveness of these matrix degradation inhibitors are necessary [45].

### 5.8. Biomaterials Used for Drug Delivery in OA

The local treatment of OA using intra-articular injections has several limitations. Circulation easily and quickly removes small molecular drugs. Moreover, a crystal suspension would be formed in the intra-articular space, leading to the risk of crystal deposition and crystal synovitis. For these reasons, a suitable drug delivery system is needed to improve the pharmacokinetics, reduce the adverse effects, and enhance the encapsulated drugs’ stability. Biodegradable materials have been introduced to prepare drug delivery systems for intra-articular injections. Some forms of chitosan nanodelivery platforms exist within the biomaterials used for drug delivery in OA. Another drug carrier is poly (lactic-co-glycolic) acid (PLGA), which is suitable for a wide range of biomolecules and to control the release of substances. Poly (N-isopropylacrylamide) (pNiPAM) is a thermoresponsive polymer for multiple applications that is used to deliver some peptides. It can directly deliver loaded peptides to the target sites and function there [58].

Polysaccharides such as chitosan, chondroitin sulphate, and hyaluronic acid have been applied in OA treatment, as mentioned previously in Section 5.2. These materials function not as carriers but as macromolecule drugs. For example, an HA-based drug delivery system displayed promising results. Therefore, the drug-in-drug concept seems to give more benefits for OA symptom relief. Synthetic polymers also provided good solubility, sustained release, and prolonged retention of the applied drug [58] (Table 2).

## 6. Conclusions and Future Perspectives

This review aimed to discuss the pathomechanisms of OA, with an emphasis on the role of cellular and humoral immune reactions in the development of the disease. The classification of OA into five phenotypes was also proposed. OA is considered to be a disease with a complex multifactorial etiology for which there is no effective therapy and therefore requires different animal models to study different bases of disease mechanisms. For this reason, the classification of several animal models, with an indication of their advantages and disadvantages, has been described in order to simplify the selection of the best model for research. This review also presented advances in the treatment of OA, i.e., pharmacological therapy, the use of various blood-derived products, surgical strategies, cell-based therapies, tissue engineering, gene therapy, and the application of biomaterials for cartilage regeneration and drug delivery. The new OA treatments, such as the application of biological agents and chemotherapeutic drugs, show better efficacy and fewer adverse reactions and seem to be more promising than traditional OA therapies. Regenerative therapy is a novel approach with the potential to restore the normal structure and function of damaged cartilage. Although current pharmacologic and regenerative therapies show excellent promise, limitations remain. New therapies may be developed by evaluating more therapeutic targets and procedures. The emerging targets confirmed in preclinical animal studies evoke a particular need to develop the most suitable animal models. The current interest in stem cell therapy can shift to other elements, including exosomes or small molecules, after clinical trials.

## Figures and Tables

**Table 1:** Summary of the advantages and disadvantages of the most popular types of animal models.

Species	Advantages	Disadvantages
Mouse	The short period between induction and the onset of disease symptoms Possible genetic modification Low doses of drugs The complete genome is known. Small living space Easy manipulations	Limited amount of tissue Limited clinical and radiological studies Inability to test for pain
Rat	Benefits listed in mice Showing pain symptoms	Small joint size Tissue and fluid volume are limited (but greater than in mice). Limited diagnostic procedures
Guinea pig	Spontaneous onset of disease in a large number of individuals Ease of maintenance in animal facilities Easy manipulations	Small size No full genome information
Rabbit	Surgical procedures are possible Small living space Easy manipulations	Incomplete disease mapping in relation to humans
Dog	Large model The possibility of training Complete genome is known (Beagle). Easy access for intra-articular therapies	Emotional and cultural controversies in many countries Genetic variation
Cat	Large model and availability of fluid and tissues for testing Complete genome is known. Possible intra-articular therapy	Emotional and cultural controversies in many countries Costly maintenance Genetic variation Different pathways of drug metabolism in relation to humans
Horse	Large model Cartilage thickness similar to humans	Economic aspect The requirement for special facilities to perform surgical procedures
Sheep	Cartilage thickness similar to humans Easy access to the joint High availability of models Availability of genetically modified strains	No full genome information available The presence of a multichamber stomach—no possibility of oral therapies
Primates	Genetically similar to humans Joint size similar to humans Possible analysis of all parts of the joint Genomes are known for some species. Possible intra-articular therapy	There is no information on the genomes of some species. Expensive research Difficult and costly model acquisition and maintenance Emotional and cultural controversies in many countries

**Table 2:** Treatment of osteoarthritis.

Treatment Regimes	Examples
Pharmacological	Growth factors, slow-acting chondroprotective drugs, metformin
Blood-derived products	PRP, hyperacute serum, platelet lysate autologous conditioned serum, autologous protein solution
Surgical	Autologous osteochondral transplantation, autologous chondrocyte implantation, osteochondral allograft transplantation, costal cartilage grafts
Cell-based therapy	Chondrocytes, mesenchymal stem cells, exosomes, pluripotent stem cells
Tissue engineering	Endogenous cell homing, cell-based and cell-free scaffolds
Gene therapy	TissueGene-C based on TGF-β gene therapy
Natural and synthetic biomaterials	Hydrogels for cartilage regeneration
Biomaterials used for drug delivery	Chitosan nanodelivery platforms

## Data Availability

Not applicable.
